# Enhancing Time Synchronization Support in Wireless Sensor Networks

**DOI:** 10.3390/s17122956

**Published:** 2017-12-20

**Authors:** Leandro Tavares Bruscato, Tales Heimfarth, Edison Pignaton de Freitas

**Affiliations:** 1 Electrical Engineering Graduate Program, Federal University of Rio Grande do Sul, Porto Alegre 90035-190, Brazil; leobrus@gmail.com; 2 Computer Science Department, Federal University of Lavras, Lavras 37200-000, Brazil; tales@dcc.ufla.br

**Keywords:** clock prediction, internet of things, low-power sensors, real-time clock, self-correcting mechanism, time synchronization, wireless sensor networks

## Abstract

With the emerging Internet of Things (IoT) technology becoming reality, a number of applications are being proposed. Several of these applications are highly dependent on wireless sensor networks (WSN) to acquire data from the surrounding environment. In order to be really useful for most of applications, the acquired data must be coherent in terms of the time in which they are acquired, which implies that the entire sensor network presents a certain level of time synchronization. Moreover, to efficiently exchange and forward data, many communication protocols used in WSN rely also on time synchronization among the sensor nodes. Observing the importance in complying with this need for time synchronization, this work focuses on the second synchronization problem, proposing, implementing and testing a time synchronization service for low-power WSN using low frequency real-time clocks in each node. To implement this service, three algorithms based on different strategies are proposed: one based on an auto-correction approach, the second based on a prediction mechanism, while the third uses an analytical correction mechanism. Their goal is the same, i.e., to make the clocks of the sensor nodes converge as quickly as possible and then to keep them most similar as possible. This goal comes along with the requirement to keep low energy consumption. Differently from other works in the literature, the proposal here is independent of any specific protocol, i.e., it may be adapted to be used in different protocols. Moreover, it explores the minimum number of synchronization messages by means of a smart clock update strategy, allowing the trade-off between the desired level of synchronization and the associated energy consumption. Experimental results, which includes data acquired from simulations and testbed deployments, provide evidence of the success in meeting this goal, as well as providing means to compare these three approaches considering the best synchronization results and their costs in terms of energy consumption.

## 1. Introduction

Society is facing the emergence of new concepts such as the Internet of Things (IoT) and its applications in domains like Industry 4.0 and Smart Cities. These new technologies and applications are responsible for a real revolution in the way data is acquired and information is processed and used to provide a myriad of useful services [1]. An important part of this complex scenario is the usage of wireless sensor networks (WSNs) that are the primary responsible for the data acquisition for the higher-end (application) systems. An important aspect related to data acquisition is the time coherence among data collected by different sensor nodes when they come to be processed or fused to compose a set of information that will be further used by higher-level applications. Particularly, this aspect is very sensitive, as wrong time–correlated data may imply erroneous information, which may cause the delivery of wrong service or even system failures. Thus, there is a clear need for a certain degree of time synchronization among these data so that they can be corrected correlated, thus being useful.

This issue about timing among the collected data can be related to the data acquisition in itself, i.e., data that are collected in different moments while they should be collected simultaneously, but also due to communication problems among the different devices and sensors composing the system. In fact, the communication itself depends on time synchronization, and the study here presented focuses on this particular aspect of time synchronization [2].

Many of the communication protocols used in IoT and WSN context usually rely on Time Division Multiple Access (TDMA) as the access method for shared medium networks, as for example in the O-MAC protocol presented in [3]. Therefore, time synchronization plays an important role in the working operation of WSN [4].

A WSN may also use synchronization for power mode energy savings, reducing collisions and scheduling for directional antenna reception. Low power consumption in sensor networks is necessary to make the system longevous. Particularly related to this aspect, it is important to notice that depending on their applications, some WSNs are intended to be deployed in harsh or difficult to access locations, such as a battlefield, an area with dangerous substances, or simply too large areas. Thus, it may be prohibitive or even impossible to just replace depleted batteries. Regarding this issue and observing that communication is a well-known power-hungry task, it means that it would be beneficial to avoid unnecessary retransmissions, which would reduce the waste of energy resources. Currently, like some of those contained in the IEEE 802.15.4 standard and the Bluetooth Low Energy, many low-power wireless communication protocols employ duty cycle scheduling, switching between active and idle modes, which is a feature that relies on clock synchronization of the nodes in the network [1].

However, WSN shows some unique characteristics that make it difficult to apply traditional network clock synchronization approaches [2]. One of the main sources of errors in clock synchronization in WSN is the non-deterministic random time delay for a message transfer between two nodes [5].

Understanding the importance of time synchronization for WSN and the challenge to achieve an efficient solution for this issue, this paper continues the studies started in [6], proposing methods to achieve time synchronization in WSNs, combining and adapting basic time synchronization methods found in the literature. The beginning of this work reported in [6] highlighted the main problems related to the overhead in number of synchronizing messages and complexity of traditional approaches applied to commercial off-the-shelf (COTS) wireless sensor nodes. Moreover, this previous work provided the direction of possible solutions for these problems using self-correction as a promising strategy for clock synchronization in WSNs. Based on these findings, the current paper reports the complete development of three different approaches for time synchronization in WSNs exploring clock estimation and smart synchronizing messages reception to enhance the synchronization with a low energy cost. The performed experiments compare the proposed approaches, showing that the one performing analytical clock prediction achieves the best results. 

The remainder of this paper is organized as follows: Section 2 presents related work. Section 3 discusses the synchronization problem and the existing basic methods to handle it. Section 4 presents the three proposed approaches, while Section 5 presents details about the clock adjustment. Section 6 presents the performed simulation-based experiments, while Section 7 presents the deployed testbed and discusses the obtained results. Finally, Section 8 concludes the paper providing directions for future work.

## 2. Related Work

Time synchronization is an important issue in Wireless sensor networks, and it has been tackled in several articles. The Timing-Sync Protocol for Sensor Networks (TPSN) is an important method presented in [7]. In this approach, a spanning tree is created, and the synchronization is realized through the hierarchical structure through the time sync packet, synchronization pulse, and ack. The procedure has two phases: first, the hierarchical structure is constructed and then the synchronization is realized. This differ from our approach due to the necessity of a hierarchical structure. Moreover, in this work, an analytical correction is proposed to reduce the transmissions to a minimum necessary.

Another well-known approach is presented in [8]. Instead of synchronizing a time server with receivers, the protocol provides synchronization among receivers that are in the range of the sender’s broadcast. This process improves time accuracy, due to the absence of the sender’s non-determinism. The article also presents an algorithm for federating clocks across broadcast domains. An estimation of the clock skew is done by using a least-squares linear regression. Multi-hop synchronization relies on gateway nodes. The drawback of the approach is necessity of a high number of messages, which may result in collisions and higher energy consumption.

In the Flooding Time Synchronization Protocol (FTSP) [9], the synchronization information is inserted in the packet as it is ready to be delivered to reduce non-determinism (MAC layer time stamping). A root node is dynamic elected to be the initiator of the synchronization process. A timestamp tuple is created at the receiver (global time, local time). These tuples are called time-stamps. For multi-hoping, differently from the TPSN, it constructs an ad hoc structure for time dissemination. This improves the robustness of the algorithm. Multiple time-stamps are stored in order to reduce jitter in interrupt handling. A linear regression prediction error is used to estimate global time. This approach requires modification of MAC layer, differently from our proposal.

There are also different approaches that are tailored for a specific protocol. For example, [10,11,12] target a protocol of the 802.15.14 standard. Our approach is platform-independent.

The work presented in [13] focuses on the time synchronization in underwater acoustic mobile sensor networks (UAMSNs). The work reports the development of an RF-based algorithm, which is bidirectional. In the first phase, it estimates the clock skew in relation to reference. Then, in the second phase, it estimates the delay between unsynchronized nodes. This algorithm implements a method of prediction designing propagation delays.

Focused on scalability and power consumption, the work presented in [14] proposed an algorithm concerned with network flooding, reporting the development of a timing method that does not overload the network with flooding messages.

Aiming at the energy savings in a wireless sensor network based on the IEEE-802.15.4 standard, the work presented in [15] reports a hybrid approach, combining the advantages of TDMA and Hopping Spread Spectrum (FHSS). Since the non-determinism of the temporal ballast is removed, the frequency difference between the local clock and the reference clock is often adjusted.

The work reported in [16] proposed a method called PulseSync, an efficient and scalable clock synchronization protocol that makes a series of measurements to determine the distance with the normal distribution. Despite the positive results achieved by the authors with their approach, the adopted assumptions are maybe too optimistic compared to those used in most of the literature (as they even recognize). Thus, it is questionable if their approach is robust in very dynamic scenarios in which those assumptions may not hold.

The work presented in [17] proposes a consensus algorithm to provide a time synchronization solution for resource constrained sensor nodes, exploring stochastic clock models. However, the accumulated forecast errors require constant resynchronization depending on the communication delay.

In [18], the authors propose an alternative autoregressive method of estimating the model parameters for time synchronization in WSNs. With this method and using generalized least squares, they estimate the relative offset and skew parameters. The performed simulations demonstrate that the method works for direct neighbor nodes, but there is no evidence that the accumulate error in multi-hop clock adjustments is kept low.

The authors in [19] propose a dynamic stochastic time synchronization scheme for WSN, which uses a Kalman filter formulation to track the clock evolution of oscillators and achieve synchrony to a central time reference. Despite targeting resource constrained nodes and presenting positive results with a testbed deployment, the complexity of the Kalman filter formulation may node be adequate to sensor nodes that have severe computation limitations, which corroborates with the authors suggestions for future work in extending the experiments to confirm the acquired results.

Observing the advancements achieved so far reported in the literature and the opportunities for enhancements, this work aims to present the effort in designing a synchronizing algorithm that addresses the problems in the previous works, namely diminishing the need for synchronization messages, diminishing the associated overhead in predictions, and providing a solution that can address dynamic operating conditions in multi-hop synchronization. The goal is to achieve a self-correcting algorithm that is adaptive for various reference clock transmission time intervals, focusing on the WSN needs for real-time IoT applications using COTS based on IEEE 802.15.4. Thus, it is possible that the contribution of this paper is based on platform/protocol independent proposal that allows the WSN developer tradeoff between synchronization precision and energy consumption, which is different from other works found in the literature that do not focus on addressing both aspects. Moreover, a complete multi-hop simulation and testbed-based experiments campaign were presented to validate the proposal, which is also not found in many related works.

## 3. Background on Time Synchronization

### 3.1. Clock Synchronization

The main types of clock synchronization are [5]

(1) Global clock: The Universal Time Coordinated (UTC) is the time standard commonly used across the world. Traditional Internet clock synchronization algorithms use the UTC to maintain this global time in all computer systems. This precision of the global time is maintained by an atomic clock. However, maintaining this synchronization in sensor networks is significantly harder, imposing a prohibitive communication overhead.
(2) Relative clock: This is the idea of relative time within the sensor network. Each node synchronizes with every other node with a given time information, which might be different from UTC. This current work uses this model to build up the proposed solution.
(3) Physical ordering: This model is used to order the events of processes in a system that shares the same clock or has a shared memory.
(4) Relative notion of time: The time can also be accorded between nodes, without using a real-time clock, but using a logical time. This model does not need to match with physical clock, i.e., when two nodes establish a connection, this moment is set as a logical time “zero”, and from this moment onward, each unit of time is incremented by both nodes, and thus a synchronism is established.

As it will be better explained, the proposal presented in this work uses models 2 and 4 before the synchronization to agree in the notion of time so that it is possible to infer when it will be the next clock transmission. Models 2 and 3 are used after the network is synchronized to perform task scheduling with the relative clock to keep the achieved synchronization.

### 3.2. Delay Component in the Synchronization Error

According to [5], the most important source of error to be considered in time synchronization systems is the non-determinism due to delay. A list of five of these source of errors is found in [20], and [21] adds the variation in quartz crystal errors to this list, as follows:

- Send time: The time used at the sender to construct the message and transfer it to the network layer for transmission [22].
- Access time: The time incurred in the MAC layer waiting for access to the transmission channel. The access time depends on the employed MAC scheme. The TDMA must wait for a specific time slot before transmission. This error depends on the network traffic and the capability of the running MAC protocol [13].
- Transmission and reception time: The delay taken by both the transmitter and receiver to send/receive the message bit-by-bit at the physical layer. It is possible to determine this delay using the packet size of the message and the baud rate of the transmissions [22].
- Propagation delay time: The delay between transmission and reception. This delay includes retransmission time due to errors. This con-siders the occurrence of errors related to the link stability, the distance between sender and receiver, among other factors [13].
- Receive time: The time spent at the network interface of the receiver node to process the incoming message and to notify the application layer of the host. Its characteristics are similar to that of the sending time, but at the receiver side.
- Quartz Crystal: As defined in [21] and studied in [23], this source of error is related to the accuracy of a single crystal of quartz. Different units of the same equipment manufactured in the same lot may have different clocks. Even the measured time can be wrong, and factors such as temperature and lifetime can affect the measurement of time. It is possible to conclude that the physical clock is inaccurate.

## 4. Time Synchronization in Wireless Sensor Networks

### 4.1. Distinguishing Characteristics of Wireless Sensor Networks

WSN have specific characteristics that are very different from ordinary computer networks. Thus, other concerns must be considered while working with WSN, which are summarized as follows [22]:

- Energy efficiency: Every protocol designed for wireless sensor networks have to consider the limited energy resources available in the sensor nodes. Knowing this, a well-designed synchronization technique should aim to minimize the number of transmissions, as communication is one of the most energy consuming tasks in a sensor node.
- Scalability: Many sensor network applications require a massive number of sensor nodes that may rely on a single time reference node. Synchronization algorithms should be able to handle situations in which this concern holds.
- Accuracy: This variable may vary depending on the specific application of the sensor network. For example, for some monitoring applications, simple ordering of events may be sufficient, while for others, microsecond accuracy may be required.
- Robustness: Synchronization algorithms should be robust against link and node failures, because networks are often left unattended for long periods of time in possibly hostile environments.
- Lifetime: The sensor nodes can remain synchronized for just a given instant, or the synchronization may last the entire lifetime of the network.
- Scope: The scope refers to the need in providing synchronization in the whole network or only local synchronization among nodes that are spatially close.
- Cost and size: Sensor nodes are commonly required to be small and inexpensive devices. It is not an acceptable solution to the synchronization problem in WSN attaching relatively large and expensive hardware such as GPS receivers or temperature compensated clocks.

### 4.2. Definition of Clock

**Figure 1:**
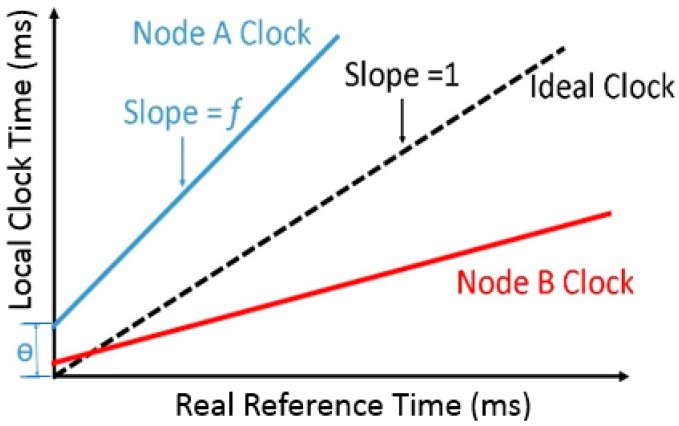
Clock model of sensor nodes.

Each sensor in a WSN has its own clock, which is based on crystal oscillators. An ideal clock of a sensor node must be set so that *C*(*t*) = *t*, where t represents the ideal or reference time. However, because of the imperfections of the clock oscillator, a clock will drift away from the ideal time even if it is initially perfectly tuned. In general, the clock function of the *i*th node is modeled as

(1) $$C_{i}\left(t\right)=\theta +f\times t,$$

where the parameters θ is called clock offset (phase difference) and f is called clock skew (frequency difference). A graphical representation of the clock model is illustrated in Figure 1. From (1), it is possible to define the clock relationship between two nodes, Node A and Node B, as follows:

(2) $$C_{B}\left(t\right)=\theta^{AB}+f^{AB}\times C_{A}\left(t\right),$$

where $\theta^{AB}$ and $f^{AB}$ stand for the relative clock offset and skew between Node A and Node B. Clearly if $\theta^{AB}=0$ and $f^{AB}=1$ the clocks are perfectly synchronized. Figure 1 represents a fast Node A, a slow Node B, and a perfect clock (dashed line) [21].

### 4.3. Synchronization Protocols

There are many proposals in the literature to address the time synchronization problem in WSNs. The work reported in [24] presents a general classification that groups the time synchronization approaches according to different criteria, as follows:

- Source of reference (standard) time: The source of the reference time may be internal, a given node inside the network, or external, a node outside the network that informs the reference time.
- Synchronization lifetime: The synchronization lifetime is a period of time in which it is required to support the consistency of clock readings. It may be permanent, in which clocks are kept synchronized during the entire network lifetime or it may be synchronization by request, in which there are periods during which no accurate information on time is required.
- Synchronization scale: It may be complete or partial, depending on the set of the network nodes that need clock correction, either all nodes or part of them, which may depend on the tasks performed by a given set of nodes during the network lifetime.
- Synchronization with shift or frequency synchronization: Frequency synchronization implies that the nodes tick at identical time intervals, while synchronization with shift implies that the nodes tick equivalent time counts.
- Synchronization of clocks and translation of time scale: Synchronization of local clocks uses either frequency synchronization or synchronization with shift, while translation of time scale translates the local time of one node to that of the other node.

According to these criteria, the proposal here presented explores the usage of an internal source of reference, with a permanent synchronization lifetime, aiming synchronize all sensor nodes in the network, with a synchronization of clocks that performs both synchronization with shift and frequency synchronization.

## 5. Proposed Approaches

### 5.1. Synchronization Algorithms

As already stated, this work proposes approaching the synchronism problem by using a time server node, which informs the other nodes about its clock, and the other nodes, in sequence, inform their clock in relation to this time server. The work explores different possibilities, proposing methods that just wait for the reference node information to adjust its clock and other which besides receiving the reference node information, also estimates the reference clock.

Waiting for the reference clock makes it possible to optimize the nodes’ behavior to be activated only when necessary. On the other hand, estimating the reference clock makes possible adjusting the clock without the reference clock in-formation.

The basis for each of these two methods were introduced in [6] and further developed and enhanced, generating a third one that outperforms the first two by introducing an analytical estimation. In this sense, the three proposed approaches represent evolutionary steps toward a synchronization algorithm that provides a better trade-off between the synchronization precision and energy consumption, as will be further explored in the results section. The rationale was to progressively combine and adapt the concepts found in the literature in using time synchronization for MAC layer protocols, probabilistic estimation of the synchronization messages’ communication delay and local and reference clock frequency actual and estimated values, to come up with a solution that meets the goals of provide the best precision at the lowest cost. The first approach in this progressive enhancing set is named Self-Correction, then the second, Clock-Prediction, and finally the third, Analytical-Correction. The first two can be understood as representatives of the majority of the approaches currently found in the literature, while the third represents a completely new approach, based on the lessons learned from the literature review and the experimentation of the first two approaches. An important assumption to be highlighted is that the approach here proposed requires that the sensor nodes be equipped with oscillators that can be digitally calibrated.

#### 5.1.1. Self-Correction

Self-Correction is a method in which a sensor node uses the received clock reference value from a time server node to approximate its own clock. This algorithm is adaptive, so it adjusts its clock in relation to the difference between its own clock and the reference one. If the discrepancy is big, the variation in the node´s clock will present a high variation.

**Figure 2:**
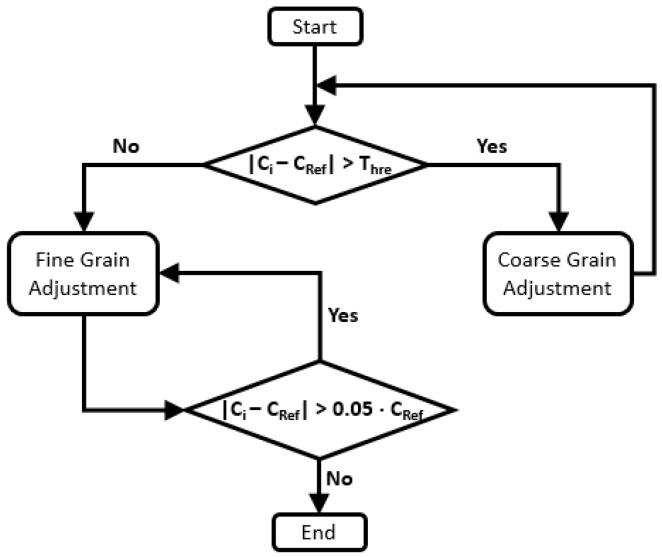
Self-Correction algorithm.

The algorithm that implements Self-Correction has a major decision to make, i.e., to decide if the difference between the reference and the local clocks is over a threshold limit in order to proceed a fine or coarse grain adjustment. According to the result of this evaluation, the fine and the coarse grain adjustment can be set to minimum or maximum values which are incremented/decremented, according to how large the difference is and if the local clock is slower or faster than the reference one. These minimum and maximum values are parameters that must be calibrated according to the specific physical clock equipping a given sensor node. If the adjustment is set to fine grain, the used value is a multiple of one hundred clock cycles—from 100 to 900 (which is also parameterized according to the specific physical clock), which is divided by an integer value that represents a counter of the increments or decrements of the fine grain adjustment. Finally, after the fine-tuning the difference between the clocks be-comes minor than 5%, the process is finished as the payback for continuing refining the result is not necessary as it will soon need to run the next adjustment. Figure 2 summarizes this algorithm.

#### 5.1.2. Clock-Prediction

The clock prediction analyzes the synchronization messages to identify the pattern of the temporal evolution. In many cases the synchronization message does not occupy the whole package. This way, it is possible to take advantage of the same package to send the information about the time interval between two transmissions together with the clock information. In possession of this information, this algorithm predicts the value of the reference clock, thus improving the accuracy of the time for larger intervals. This algorithm was inspired by the work presented in [25].

Using the additional available information about the time interval, explained above, this algorithm analyzes the last transmissions to predict the value of the clock of the reference node. This makes it possible to continue adjusting the local clock during the period in which the reference node is not transmitting, i.e., during the intervals between two transmissions. As an example, given a time interval of 30 s between two transmissions, this algorithm is able to make 28 clock corrections, the first with the received value of the reference node, and the remaining 27 are with the estimated value. The reference clock is calculated as follows:

(3) $$C\_Ref_{K+1}=C\_Ref_{K}+\Delta C\_Ref_{k}$$

where

(4) $$\Delta C\_Ref_{k}=\frac{\Delta C\_Ref_{k-1}}{2}+\frac{\Delta C\_Ref_{k-2}}{4}+\frac{\Delta C\_Ref_{k-3}}{8}+\frac{\Delta C\_Ref_{k-4}}{16}+\ldots$$

The receiver node uses the same function of the self-correction to achieve the best result with the same time interval. As the synchronism is not perfect, the receiving node is able to receive a new message with at least 5% latency (assumed as a tolerable margin of error in the time interval).

#### 5.1.3. Analytical-Correction

To further improve the precision of the Clock-Prediction algorithm and thereby reduce the transmissions to a minimum necessary, it was developed the Analytical-Correction algorithm inspired by ideas presented [26,27]. It works as follows: the reference node informs the slope of its time function so that the receiving device adapts its own to improve the shape of the function that represents the evolution of its clock. This means that the sender node is considered as the reference one, and the receiving node synchronizes according to this reference. The algorithm tries to match the clocks as fast as possible and then tries to have the same slope as the reference node.

The clock adjustment to support this third approach uses more information about the reference node: in addition to transmitted clock information, it also uses information about the inclination of the clock function, which is sent by the reference node so that to the receiver node can calculate the best fit for the clock function. In this way, the clock of the synchronizing node converges faster, as this method looks for the best inclination so that the clocks are as close as possible, as described in (5), which find the minimal difference between the projected RTC of reference node and the future possible node’s RTC. If multi-hops are considered, the synchronizing node can modify its slope, to fit its referencing node, and transmit this new value to serve as reference to the next node in a chain.

(5) $$Min\left(\left|C\_Projected-C_{ID}+t*C\_Tab\left[i\right]\left[j\right]\right|\right)$$

*Tune* = [*i*,*j*] (6)

where *Tune* is the adjust of Clock, $C_{ID}$ is the device clock, *C_Tab* is a table that stores all possible values for each tuning option, *t* is the time interval, and the C_Projected is define by

(7) $$C\_Projected=C_{ID-1}+t*C\_Tab_{ID-1}\left[i\right]\left[j\right]$$

### 5.2. Reference and Synchronizing Nodes Operation Modes

In order to implement the above presented synchronization algorithms, both the Reference and Synchronizing nodes need to match in relation to their operation modes. The details about their operation modes are presented in the following.

#### 5.2.1. The Reference Node

This node has the function of sending its clock to synchronize the other nodes. For this, it sends its message early in its sending period (the time interval between two transmissions) and waits until the end of this period. Until the next transmission, the reference node may switch to idle mode or perform another task.

#### 5.2.2. The Synchronizing Node

As the goal is to establish communication with the referencing node, three different modes for the synchronization node operation were defined. The first mode is always able to receive the synchronization message, i.e., the node is always on and waiting for the synchronizing message. This mode is used when the time of the next transmissions of the synchronizing message is unknown. This is the case with the highest energy consumption.

The second mode has the goal of making the synchronizing nodes more efficient, so after receiving the synchronization message, they can perform another task or switch to idle mode at the end of the period. This is possible, because the synchronization message also contains the information about the transmission period (for the Clock-Prediction and Analytic-Correction approaches) and with that information, the synchronizing nodes can be programmed to weak-up for the next transmission. This feature plus the fact that synchronization messages can be sent in large intervals in the proposed protocol brought a considerable energy saving. Other protocols, as the TPSN [7], RBS [8] and FTSP [9] do not address explicitly the combination in this manner. Therefore, it is possible to expect that the proposed protocol can maintain clock synchronization in combination with duty-cycle. This is a feature that allows less energy to be employed. In fact, the presented protocol is more concerned at achieving a time synchronization with sufficient precision efficiently, focusing on saving energy with less message exchanges and programmed sync periods.

The third mode tries to improve the synchronization performance, analyzing the difference between the last transmissions of the reference node. Then, it projects a value of the reference clock, which is used to adjust the local clock between the transmissions of the reference node.

## 6. Simulation

Simulations were performed in order to assess the efficiency of each of the proposed approaches, aiming to compare them in relation to the synchronism of the network and the time spent to achieve the synchronism in single and multi-hop communication. It is fundamental to make this comparison as fair as possible, and for this, it is necessary that all initial conditions, server clock, synchronizing clock and clock configuration, must be the same in all cases. The simulations were implemented in Python modeling the behavior and the architecture of a real wireless sensor node device, the FreeScale MC1322x [27]. The algorithms implementing the proposed methods described above work as follows: For the Self-Correction, the clock adjustment occurs only once between the transmissions of two synchronization messages. The receiver node decides about how to adjust its clock based on the different between its own clock and the received information. If the difference is greater than 10 times the oscillation amplitude, it will consider if the its clock is faster or slower and proceed to evaluate if a fine or coarse tune is needed. If the difference is greater than the limits imposed by the sensor node being used (which depends on the manufacturer’s specifications) for the fine tuning, a coarse-grain one is executed. The second proposed method, the Clock-Prediction, uses the same algorithm described above, but it performs more than just one correction between two synchronization messages transmissions. This is possible because the synchronizing node calculates the slope of the reference node based on the average of the values received in the previous received synchronizing messages. In the third method, the Analytical-Correction, the reference node sends not only the information about its clock, but also the slope, i.e., the evolution of its clock. With this information, the receiving node can perform successive corrections in its own node in order to approximate its slope to the received one. The complete code used to implement the performed simulations is available for download. In the following link, the complete code used to implement the simulations can be downloaded: https://drive.google.com/open?id=1MyStf9-ZBBj77CyJ9jh0HLdHq0ZwOCja.

### 6.1. Single-Hop

**Figure 3:**
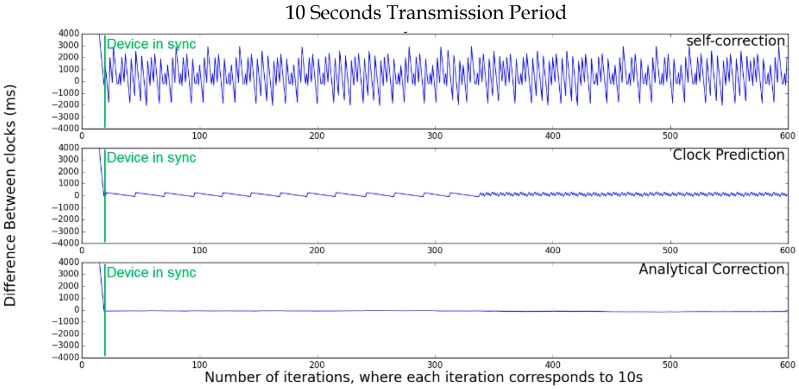
Single-hop simulation.

This simulation was designed with a single server node that sends a timestamp to one client node. The client (synchronizing) node uses its sync algorithm, adjusting its clock as necessary. The server boots with the value of 20 s and the client nodes half a second, simulating a server that is activated long before the clients. Figure 3 presents the simulation of the clock values of the synchronizing node for each algorithm. It is possible to see in the graphs depicted in this figure that all methods converge at the same instant, with 20 iterations (highlighted in the graphs by a line labeled “Device in sync”), and the precision of the self-correction is the worst of all. It is also possible to observe in Figure 3 that the accuracy of the Clock-Prediction algorithm is much better than Self-Correction, but the best performance is achieved by the Analytical-Correction.

### 6.2. Multi-Hop

**Figure 4:**
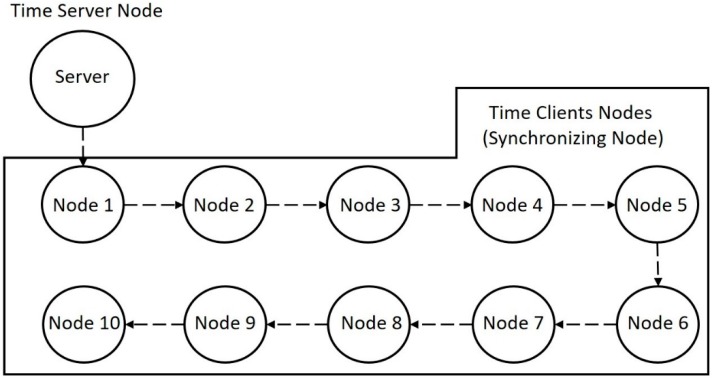
Multi-hop simulation design. To simplify the graphical presentation of the simulation results, only the measured results for nodes 1, 2, 5, and 10 are displayed. Since nodes 3 and 4 have intermediate values between nodes 2 and 5, and nodes 6, 7, 8, and 9 are intermediate nodes between 5 and 10, this simplification can be made assuming that the errors are cumulative. Thus, the provided results are representative to the whole network.

These simulations were designed to test the chain synchronism, i.e., the synchronization that is performed considering multi-hops between the time server and the synchronizing nodes. The server node sends its timestamp to the first node (the closest one), which then transmits to the second, and so on until reaching the 10th node. With the same initial values of the single-hop simulation, each node receives the timestamp of its reference node, i.e., the previous node in the chain, as represented in Figure 4.

**Figure 5:**
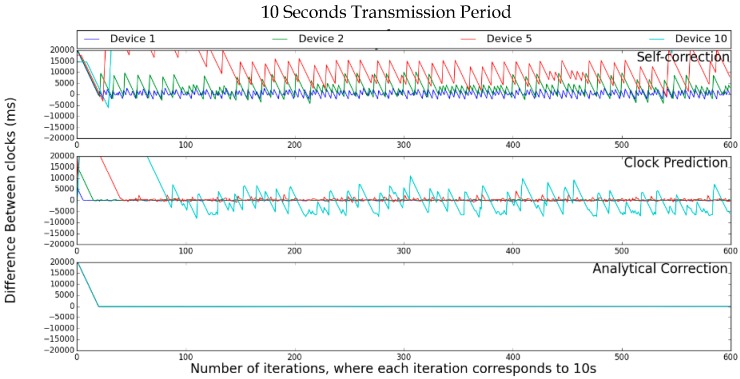
Multi-hop simulation.

Figure 5 presents the simulation results of the clock values for the chain synchronization with each algorithm. It is possible to observe that, with the Self-Correction, nodes 5 and 10 do not synchronize after 600 iterations (and possibly will never reach synchronism at all). With the Clock-Prediction, until device 5, the network reaches the synchronism in less than 40 iterations, but node 10 still has not reached the synchronism with 600 iterations. The Analytical-Correction presented the best results, as all nodes synchronized in less than 20 iterations and remained so until for the rest of the test.

It is important to highlight that these simulations present characteristics that are not the same as those observed in real world devices. As an example, the clock behavior for all simulated devices is considered equal. In fact, real-world devices have different internal clocks that behave differently, as highlighted in Section 3.2. However, random variations were added to the clock value based on the maximum difference between the real nodes used in the testbed, in order to make the simulations closer to the real deployments. Another characteristic that stands out is the temporal evolution of the clocks. Using real devices, this evolution happens in a continuous and linear way, but in this simulation, all the temporal evolutions occurred in an abrupt and fragmented form, since there was a discrete function that updated all the values of each simulated device. Thus, besides the information provided by the simulation results, additional experiments with real-word devices were necessary to validate the proposed synchronization methods.

## 7. Testbed Experiments

Testbed experiments were performed to assess the efficiency of each of the proposed approaches. The used equipment to perform these experiments is presented in the following, and then the setup of the performed experiments is described. The algorithms are the same as those used in the simulations. The complete code used to deploy the testbeds is available for download. The complete code used in the deployment of the testbeds can be downloaded at https://drive.google.com/open?id=1YA2ztOXBUJIhBS1EGBSDcAODxORE4uPP.

### 7.1. The Sensor Node

The wireless sensor nodes devices used for the performed experiments are based on the NXP^®^ Semiconductor’s third-generation 2.4 GHz IEEE^®^ 802.15.4 platform, namely Freescale MC13224V. This platform is equipped with a programmable 32-bit ARM7 core–based microcontroller unit. It uses a crystal oscillator with a typical frequency ranging from of 2 to 25 kHz. This oscillator has two ways of adjustment, i.e., the coarse tune that can change each step loading by 1 pF, and the fine tune that changes each step loading by 160 fF [27]. This oscillator is digitally calibrated by changing the value stored in its memory address (bits 4–8 of for the fine tune and bits 9–12 for the coarse-grain tune).

To support C coding and programming, IAR Embedded Workbench software was used together with a Segger J-Link, a debug probe containing a JTAG interface.

A connectivity test demo comes preprogrammed in the sensor node. This software was adapted to match the purposes of the designed experiments.

### 7.2. Experiments Setup

The performed experiments are divided into two types to match the two different simulations presented in Section 6: (i) client-server and (ii) chain clock propagation. In the client-server, one node acts as the time server, which provides the Reference Clock, and the other acts as the receiving client node (the synchronizing node), which has the goal to synchronize its clock with the reference server one.

For the client-server experiments, four different transmission periods (time interval in which the time server sends Sync message) were tested for each one of the proposed approaches, i.e., 1, 10, 30, and 60 s. The time server node used the loop sending one transmission in each period and the synchronizing node used the three synchronization algorithms: Self-Correction, Clock-Prediction, and Analytical-Correction. For each setup, 120 iterations were performed.

**Figure 6:**
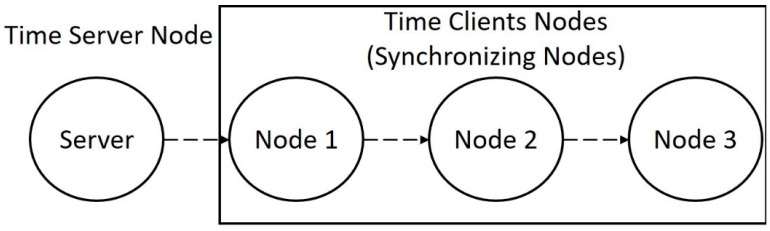
Test layout of many client and server network.

For the chain synchronization, a set of four nodes was used in which there is a time server that transmits its current clock to the first node. Then this first one transmits to the second, and finally, the second transmits to the third. This setup is presented in Figure 6. In this case, the analysis is made in relation to the latency of the network. The goal of these performed experiments is to measure the difference between two clocks after a long period of time to check whether they are still partially synchronized.

### 7.3. Testbed Results

**Figure 7:**
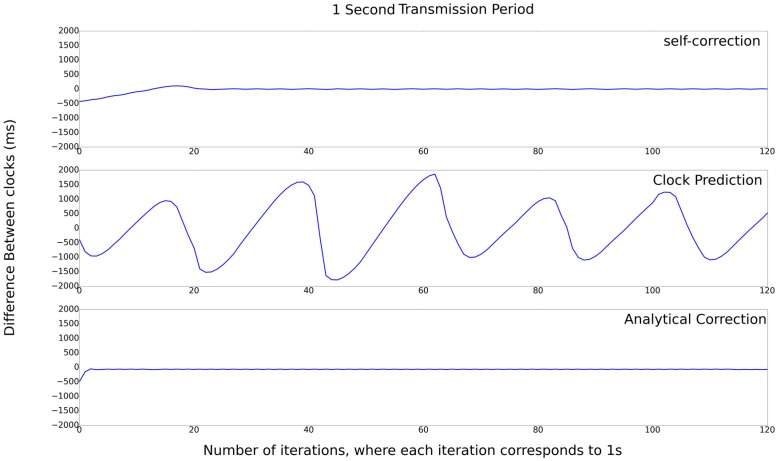
Set of experiments with 1 s transmission period.

The results for the first set of experiments are presented in Figure 7, which presents the difference between the server and the client nodes for the first type of experiment (client-server). Analyzing this figure, it is possible to observe that the results of Self-Correction and Analytical-Correction algorithm are very close to 0, and that the Clock-Prediction algorithm presents the worst result. The Clock-Prediction algorithm cannot converge its clock with the reference clock. Between the Self-Correction and the Analytical approaches, the latter has a slight advantage, being the best choice for this value of transmission period.

**Figure 8:**
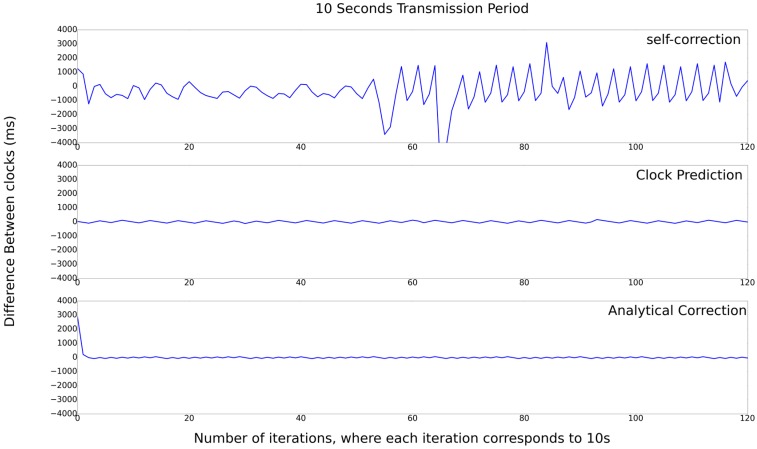
Set of experiments with 10 s transmission period.

Next, the results presented in the Figure 8 provide the same comparison presented in Figure 6, but with the transmission period of 10 s. By the results, it is possible to observe that the difference ranges between plus and minus 4000. As expected, the higher the transmission interval is, the worse is the timing accuracy. In this case, the self-correction algorithm presents the worst results, with an error greater than 4 s, and the Analytical-Correction algorithm is a little better than Clock-Prediction algorithm, with a maximum error of 200 and 162, respectively. For this test, the three algorithms converged for the reference clock. For this case, the algorithm that presents the best result is Clock-Prediction.

**Figure 9:**
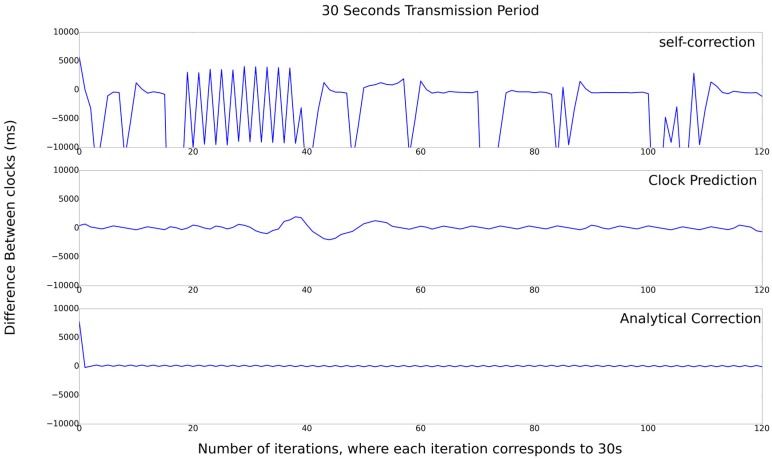
Set of experiments with 30 s transmission period.

In Figure 9, the results for the 30 s transmission period is depicted. It is possible to observe that the difference ranges between 10,000 and −10,000, with the same crystal oscillator, having the largest time discrepancy between nodes on 10 s in the Self-Correction, more than 2 s in the Prediction-Clock, and 195 ms in the Analytical-Correction algorithm. It is clear that for this case, the algorithm that presents the best result is Analytical-Correction.

**Figure 10:**
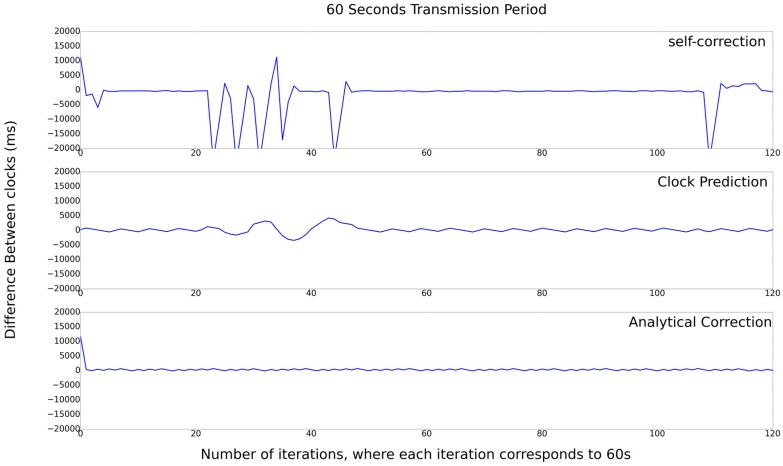
Set of experiments with 60 s transmission period.

In Figure 10, the result for the 60 s transmission period is depicted. It is possible to observe that the difference ranges between 20,000 and −20,000. In this case, the Self-Correction algorithm presents the worst result, with an error greater than 20 s. The Prediction-Clock algorithm has an error of approximately 4.2 s, and the Analytical-Correction algorithm presents an error of 200 ms, notably the best result.

**Figure 11:**
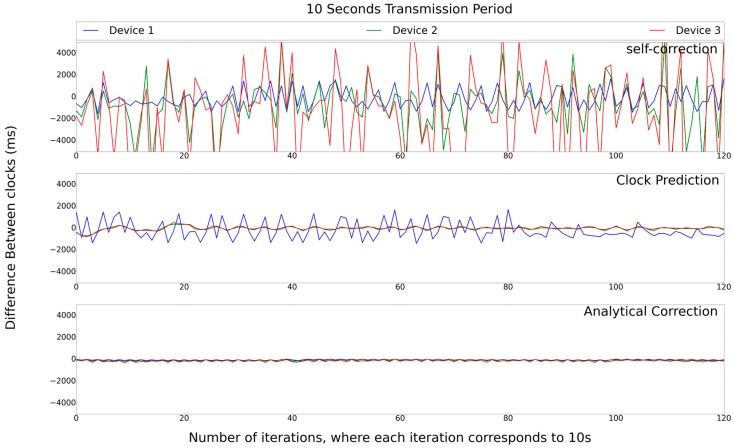
Set of experiments with chain synchronization.

The second test set assesses the chain synchronization behavior using a transmission period of 10 s and testing all the synchronized methods proposed in this work. As Figure 11 shows, only one of the algorithms has a synchronization error greater than 4 s in the fourth node, which is the Self-Correction. In the Clock-Prediction algorithm, the network remains synchronized, even if the second node has a synchronization error of 2 s, but the other nodes soften the clock of node 2. Thus, the error is very close to zero. For the Analytical-Correction algorithm, all nodes have a close to zero error, and it is certainly the best choice.

According to the definitions in Section 4.2, two devices are considered synchronized if they have offset equal to 0, clocks equal, and have clock skew equal to 1, besides the same slope for the clock function. Then, from this definition, it is possible to state that the network remained synchronized for all cases when the Analytical-Correction algorithm was used, because the difference was very close to zero, and the clock function formed almost a straight line having a derivative equal to 1.

**Figure 12:**
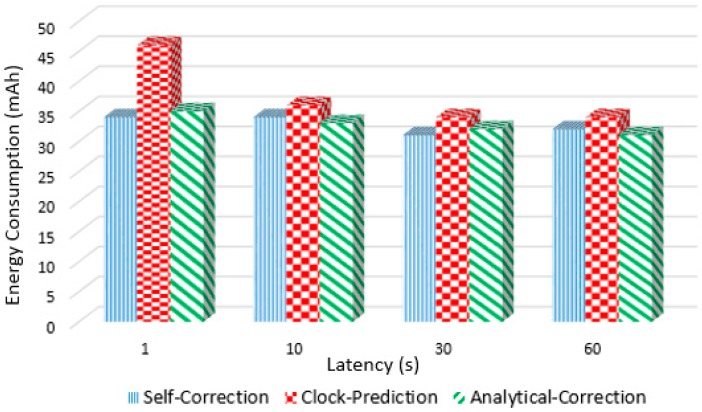
Energy consumption results during 1 h.

Another important analysis to be made is in relation to the energy consumption. In order to identify the difference between the three proposed methods (Self-correction, Clock-Prediction, and Analytical-Correction), the energy consumption associated to each of them was analyzed. Figure 12 presents the average of obtained results for all tested transmission periods in the chain clock propagation scenario, in which the energy consumption is measured in mAh. In all cases the duration of the performed tests was of one hour and to measure this consumption, an external meter was used. As the meter has the smaller scale in mAh, it is possible to assume 1 mAh of lack of precision. It is possible to observe that the Clock-Prediction presented the higher energy consumption. These results, analyzed together with the clock synchronization ones presented above, allows to state that the Analytical-Correction is the most suitable approach, presenting the best results in terms of synchronization and energy consumption. The other two present a trade-off between the two metrics, while the Self-Correction has lower energy consumption compared to Clock-Prediction, while this last one presents better clock synchronization results.

## 8. Conclusions

This work introduces three approaches to establish temporal synchronism in low power wireless sensor networks. This goal is very important, since the synchronization of sensor nodes is important in various applications, for example, in order to retain temporal correlations of sensor readings at different points of the network. In this work, different concepts related to the topic and found in the literature are studied, and three approaches are developed based on the existing concepts. The presented protocols are Self-Correction, Clock-Prediction, and Analytical-Correction. In Self-Correction, a sensor node uses the received clock reference from a time server to adjust its clock. Clock-Prediction employs latency and clock information to predict the value of the reference clock. In the Analytical-Correction approach, the slope of its time function is informed in order to allow adaptation of the shape of the function of its own clock. As can be observed in the reported results, the best proposed solution is Analytical-Correction, which combines information from the reference node with those of an analytical calculation method to achieve the synchronization as fast as possible and keep it once achieved. For the development of this solution, specific characteristics of WSN were considered: energy restriction, scalability, and precision. Thus, it manages a good result in terms of the trade-off between the required synchronization and an acceptable overhead in terms of power consumption due to communication. As an adaptive system, it runs when necessary, maintaining low power consumption while keeping a high synchronization between the sensor nodes.

The proposed work provides large intervals of synchronization messages with combination of the duty-cycle mechanism, focusing on an adequate synchronization with low energy consumption. In the context of synchronization protocols for WSN several others are concerned with high precision, which is not necessary for all kinds of applications. Moreover, some other protocols are strictly linked to the MAC layer, which is not the case for the proposal here presented.

The effectiveness of the three proposed methods were assessed through a simulation and experiments with Freescale sensor node platform. The Analytical-Correction algorithm provides the best performance among the three methods, having a one-second error for a 60 s transmission period. When the multi-hop synchronization test is taken in consideration, experiments showed that it had an accumulated error of less than 300 ms in the fourth node (node 3). In other words, the largest delay from node 3 to the server was less than 0.3 s for the tests with latency of 10 s. Analyzing the energy consumption results, the Self-Correction and the Analytical-Correction approaches have very similar results, with a slightly advantage to the last one. However, when the synchronization precision is also considered, the Analytical-Correction was the best approach.

Future works will focus in implementing the Analytical-Correction algorithm in heterogeneous sensor networks, with different sensor node devices and hence different internal clocks. This is important to make the solution suitable to IoT application based on COTS devices, which may use various appliances from different brands, which by their turn may have different internal components, including the clock. Another important direction for further investigations is about the performance degradation in face of interferences in the transmission of the synchronization messages.
